# Supplementary material for: Usefulness of atezolizumab plus bevacizumab as second-line therapy for patients with unresectable hepatocellular carcinoma
Source: PLoS One. 2024 Apr 30;19(4):e0298770. doi: 10.1371/journal.pone.0298770 (PMC11060596; doi:10.1371/journal.pone.0298770)
Supplement: S1 File — (DOCX) [file pone.0298770.s002.docx]

Study Protocol for the Evaluation of Treatment Outcomes of Atezolizumab/Bevacizumab Therapy for Unresectable Hepatocellular Carcinoma

Principal Investigator

Institution: Saitama Medical University Hospital

Department: Gastroenterology and Hepatology

Name: Satoshi Mochida

1. Objective of the study

To elucidate the efficacy of atezolizumab/bevacizumab therapy for unresectable advanced hepatocellular carcinoma.

1. Background and significance of the study

There are several treatment options for hepatocellular carcinoma, including surgery, radiofrequency ablation, transarterial chemoembolization, hepatic arterial infusion chemotherapy, molecular-targeted agents, and liver transplantation. The treatments for advanced hepatocellular carcinoma, which was diagnosed as unresectable, were limited to four molecular-targeted agents: sorafenib, regorafenib, lenvatinib, and ramucirumab. In November 2020, the atezolizumab plus bevacizumab therapy with immune checkpoint inhibitors was newly approved. Compared to sorafenib, this combination therapy has been reported to have a higher survival rate and response rate. Therefore, we evaluate the usefulness of atezolizumab plus bevacizumab based on the outcome of patients receiving these agents in our hospital.

1. Organization of the study
2. Principal investigator

Institution: Gastroenterology and Hepatology

Name: Satoshi Mochida (Professor)

1. Investigator: Listed separately
2. Research office and responsible staff (if applicable): Not applicable
3. Other external institutions (if applicable): Not applicable
4. Research methods and duration
5. Definition of study subjects:

Patients aged 20 years or older with hepatocellular carcinoma treated with atezolizumab/bevacizumab therapy in our hospital.

1. Target number of cases

Total of 85 cases at Saitama Medical University Hospital.

1. Research duration

Study period: December 1, 2020 – August 31, 2022

Overall research duration: From approval date to March 31, 2024

1. Survey/investigation items

Patient demographics (age, gender, cause of liver disease), treatment commencement date, history of hepatocellular carcinoma treatment, liver function reserve, tumor size & number, extrahepatic lesions, degree of portal vein invasion, tumor markers (AFP, PIVKA-II), thyroid function tests, other blood biochemistry tests (white blood cells, hemoglobin, platelets, AST/ALT, creatinine, albumin, total bilirubin, ammonia, PT%), urine test (urinary protein), antitumor effects, adverse events, and outcomes.

1. Statistical Methods:

The survival rate will be examined using the Kaplan-Meier method. Other items will be examined using chi-square or Fisher's exact test, Wilcoxon test, and logistic regression analysis. A p-value of <0.05 (two-sided test) will be considered statistically significant.

1. Others: Not specified
2. Public disclosure of research information

Not applicable

1. Sample & information storage

Information collected from medical records will be recorded on an external storage medium using an offline computer. The storage medium will be securely locked away. After retaining the data for 5 years post-research, it will be properly discarded, ensuring the prevention of personal data leakage.

1. Personal information protection

This clinical study is exclusively conducted at our hospital, and all data analyses are performed in-house. Hence, no personal data of the participants, such as name, ID, and date of birth, will be disclosed externally. The protection of personal data will be duly considered at the time of publication.

1. Informed consent:

As this is a retrospective study, we will not obtain informed consent from participants. However, we will disclose the study information and ensure that participants have the opportunity to refuse participation.

Information disclosure location:

Saitama Medical University Hospital IRB homepage

URL: <http://www.saitama-med.ac.jp/hospital/outline/irb.html>

1. Response to queries and concerns from participants

Contact: Department of Gastroenterology and Hepatology, Saitama Medical University Hospital

Phone number: 049-276-1198

1. Financial matters

Funding source:

Gastroenterology and Hepatology general research fund

Conflicts of interest pertaining to research:

Researchers' conflicts of interest are appropriately managed as declared to the Saitama Medical University Hospital COI Committee.


1. Reporting to the hospital director

1) Permission to conduct the research:

The principal investigator confirms that approval has been obtained from the hospital IRB and permission from the hospital director before initiating the research.

1. Amendments to the study protocol:

- Should there be any changes to the study protocol, the principal

investigator will promptly apply for a modification, obtain the hospital IRB's approval, and then secure permission from the hospital director.

1. Reporting on the research status:

The principal investigator will report the research's status to both the hospital director and the hospital IRB at least once a year.

1. Research completion:

The principal investigator will report to both the hospital director and the hospital IRB as soon as the research is completed.

1. Publication of research results

Results will be published in a journal in the field of gastroenterological disease.

1. Intellectual property rights

The outcome of this research may result in intellectual property rights. Any such rights will belong to Saitama Medical University, and the participants will have no claims to them.

Investigator List

Satoshi Mochida：Department of Gastroenterology and Hepatology (Professor)

Tomoaki Tomiya：Department of Gastroenterology and Hepatology (Professor)

Yukinori Imai：Department of Gastroenterology and Hepatology (Associate Professor)

Nobuaki Nakayama：Department of Gastroenterology and Hepatology (Associate Professor)

Suguru Mizuno：Department of Gastroenterology and Hepatology (Associate Professor)

Kayoko Sugawara：Department of Gastroenterology and Hepatology (Lecturer)

Masamitsu Nakao：Department of Gastroenterology and Hepatology (Assistant Professor)

Satsuki Ando：Department of Gastroenterology and Hepatology (Assistant Professor)

Hiroshi Uchiya：Department of Gastroenterology and Hepatology (Assistant Professor)

Yoshihito Uchida：Department of Gastroenterology and Hepatology (Assistant Professor)

Keisuke Shiokawa：Department of Gastroenterology and Hepatology (Assistant Professor)

Akira Fuchigami：Department of Gastroenterology and Hepatology (Assistant Professor)

Masashi Takano：Department of Gastroenterology and Hepatology (Assistant Professor)

Maiko Asami：Department of Gastroenterology and Hepatology (Assistant Professor)

Yoichi Saito：Department of Gastroenterology and Hepatology (Assistant Professor)

Takanobu Suzuki：Department of Gastroenterology and Hepatology (Assistant Professor)

Shinpei Yamaba：Department of Gastroenterology and Hepatology (Assistant Professor)

Hayato Uemura：Department of Gastroenterology and Hepatology (Assistant Professor)

Naoto Soma：Department of Gastroenterology and Hepatology (Assistant Professor)

Daisuke Hamada：Department of Gastroenterology and Hepatology (Assistant Professor)

Shohei Tsuji：Department of Gastroenterology and Hepatology (Assistant Professor)

Hayato Kurata：Department of Gastroenterology and Hepatology (Assistant Professor)

Rui Ushiyama：Department of Gastroenterology and Hepatology (Assistant Professor)

Aya Sato：Department of Gastroenterology and Hepatology (Assistant Professor)

Shunsuke Yamada：Department of Gastroenterology and Hepatology (Assistant Professor)

Nanase Usui：Department of Gastroenterology and Hepatology (Assistant Professor)
