# Supplementary material for: Usefulness of atezolizumab plus bevacizumab as second-line therapy for patients with unresectable hepatocellular carcinoma
Source: PLoS One. 2024 Apr 30;19(4):e0298770. doi: 10.1371/journal.pone.0298770 (PMC11060596; doi:10.1371/journal.pone.0298770)
Supplement: S2 File — (DOC) [file pone.0298770.s003.doc]

切除不能肝細胞癌に対する

アテゾリズマブ/ベバシズマブ療法の治療成績の検討

研究計画書

　　　　　　　　　　　　　　　　　　　　　　　 　研究責任者

＜研究機関名＞埼玉医科大学病院

　＜所属＞　消化器内科・肝臓内科

＜氏名＞　　　　　　　持田　智

**１.　研究の目的**

切除不能進行肝癌に対するアテゾリズマブ/ベバシズマブ療法の有効性について明らかにする。

**２.　研究の背景と意義**

肝細胞癌に対しては，手術・ラジオ波焼灼術・肝動脈化学塞栓術・肝動注療法・分子標的薬・肝移植など複数の治療法が存在し，その進行度によって治療法を決定する。今まで切除不能と診断された進行肝細胞癌に対する治療は，分子標的薬であるソラフェニブ・レゴラフェニブ・レンバチニブ・ラムシルマブの4剤であったが、2020年11月に免疫チェックポイント阻害薬を加えたアテゾリズマブ+ベバシズマブ療法が新たに承認された。アテゾリズマブ/ベバシズマブ療法は、ソラフェニブに比して高い生存率と奏効率が報告されており，進行肝癌例での治療成績の向上に寄与すると期待される。そこで，当院におけるアテゾリズマブ/ベバシズマブ療法を開始した症例の治療成績を検討することで，その有用性を評価する。

**３．研究の実施体制・組織**

１）主任研究者

　　所属　消化器内科・肝臓内科　　（役職　教授）　　　氏名　持田　智

２）研究実施者：別紙記載

３）研究事務局及び担当者（該当する場合）：該当せず

４）上記以外のデータセンターなどの外部機関（該当する場合）：該当せず

**４.　研究の方法と期間**

１）研究対象者の定義

　　当院にてアテゾリズマブ/ベバシズマブ療法を施行した20歳以上の肝細胞癌症例

２）目標症例数

　　埼玉医科大学病院における症例数　　（　85　）例

３）研究の期間

調査対象期間：　2020年　12月　1日　～　2022年　8月　31日

研究期間：　承認日　～　2024年　3月　31日

４）調査項目/検査項目

患者背景（年齢、性別、肝疾患の成因），治療開始日，肝細胞癌治療歴、肝予備能，腫瘍径・個数，肝外病変，門脈浸潤度，腫瘍マーカー（AFP、PIVKA-Ⅱ），甲状腺機能検査，その他の血液生化学検査（白血球・ヘモグロビン・血小板・AST／ALT・クレアチニン・アルブミン・総ビリルビン・アンモニア・PT％），尿検査（尿蛋白），抗腫瘍効果，有害事象，転帰

５）統計処理を行う方法

生存率をKaplan-Meier法により検討する。その他の評価項目については分割表によるχ^2^もしくはFisherの正確確率検定、Wilcoxonの検定、ロジスティック回帰分析を用いて検討する。なお、P<0.05（両側検定）を有意差ありとする。

６）その他

**５.　研究に関する情報公開について**

該当せず

**６．試料・情報の保管について**

診療録より取得した情報は、インターネットと切り離されたコンピュータを使用し外

部記憶媒体に記録させ、その記憶媒体は鍵をかけて厳重に保管する。研究終了後5年

間保管した後、個人情報の漏洩に配慮し適切に廃棄する。

**７．個人情報保護の方法**

当院単独の臨床研究であり、データの解析等もすべて当院で実施するため、研究実施中に被験者の氏名、ID、生年月日などの個人情報が、外部に出ることはない。公表時にも被検者の個人情報保護については十分に配慮する。

**８．インフォームドコンセント**

後ろ向き研究のため、被験者からインフォームドコンセントは受けないが、研究の情報を公開し、研究対象者が拒否できる機会を保障する。

　　情報公開する場所

埼玉医科大学病院ＩＲＢホームページ

　 URL: <http://www.saitama-med.ac.jp/hospital/outline/irb.html>

**９．研究に関する被験者からの相談等の対応**

以下の連絡先を相談窓口とする。

連絡先：埼玉医科大学病院　消化器内科・肝臓内科

電話番号：049-276-1198

**１０．費用に関する事項**

１）研究の資金源

消化器内科・肝臓内科 一般研究費

２）研究機関の研究に係る利益相反について

　研究者の利益相反は埼玉医科大学病院COI管理委員会に申告するなどして適正に管理されている。

**１１．病院長への報告に関する事項**

1. 研究の実施の許可：

研究責任者は、研究の実施に先立ち、本研究計画書について病院ＩＲＢの承認及び病院長の許可を得ていることを確認する。

1. 研究計画内容の変更：

研究責任者は、研究計画書内容に変更点が生じた場合は、速やかに変更申請をし、病院ＩＲＢの承認を得て、病院長の許可を得る。

1. 実施状況報告：

研究責任者は、少なくとも年に１回以上の頻度で、研究の実施状況を病院長及び病院ＩＲＢに報告する。

1. 研究終了時：

研究責任者は、研究が終了したら速やかに病院長と病院ＩＲＢに報告をする。

**１２．研究結果の公表**

消化器病関連の雑誌にて論文発表する。

**１３．知的財産権について**

　　本研究の成果により、知的財産権が生じる可能性がある。その権利は埼玉医科大学に属し、被験者に知的財産権は属さない。

＜別紙＞　研究組織　一覧

研究実施者

| 氏名 | 所属（役職） |
| --- | --- |
|
| 持田　智 | 消化器内科・肝臓内科（教授） |
| 富谷　智明 | 消化器内科・肝臓内科（教授） |
| 今井　幸紀 | 消化器内科・肝臓内科（准教授） |
| 中山　伸朗 | 消化器内科・肝臓内科（准教授） |
| 水野　卓 | 消化器内科・肝臓内科（准教授） |
| 菅原　通子 | 消化器内科・肝臓内科（講師） |
| 中尾　将光 | 消化器内科・肝臓内科（助教） |
| 安藤　さつき | 消化器内科・肝臓内科（助教） |
| 打矢　紘 | 消化器内科・肝臓内科（助教） |
| 内田　義人 | 消化器内科・肝臓内科（助教） |
| 塩川　慶典 | 消化器内科・肝臓内科（助教） |
| 渕上　彰 | 消化器内科・肝臓内科（助教） |
| 鷹野　雅史 | 消化器内科・肝臓内科（助教） |
| 浅見　真衣子 | 消化器内科・肝臓内科（助教） |
| 齊藤　陽一 | 消化器内科・肝臓内科（助教） |
| 鈴木　隆信 | 消化器内科・肝臓内科（助教） |
| 山羽　晋平 | 消化器内科・肝臓内科（助教） |
| 植村　隼人 | 消化器内科・肝臓内科（助教） |
| 相馬　直人 | 消化器内科・肝臓内科（助教） |
| 濱田　大祐 | 消化器内科・肝臓内科（助教） |
| 辻　　翔平 | 消化器内科・肝臓内科（助教） |
| 倉田　隼斗 | 消化器内科・肝臓内科（助教） |
| 牛山　叡 | 消化器内科・肝臓内科（助教） |
| 佐藤　彩 | 消化器内科・肝臓内科（助教） |
| 山田　俊介 | 消化器内科・肝臓内科（助教） |
| 碓氷　七瀬 | 消化器内科・肝臓内科（助教） |
