# Supplementary material for: Usefulness of atezolizumab plus bevacizumab as second-line therapy for patients with unresectable hepatocellular carcinoma
Source: PLoS One. 2024 Apr 30;19(4):e0298770. doi: 10.1371/journal.pone.0298770 (PMC11060596; doi:10.1371/journal.pone.0298770)
Supplement: S3 File — (PDF) [file pone.0298770.s004.pdf]

Age	Etiology	ALBI grade	BCLC	Portal vein tumor thrombosis
1	2	1	2	2
2	2	1	2	1
2	2	1	2	2
1	1	2	2	2
2	2	1	2	2
1	2	2	1	1
2	1	1	2	2
1	2	2	1	1
2	2	1	1	1
2	2	2	1	1
2	1	2	1	1
2	2	1	2	2
2	2	2	1	1
1	1	1	2	2
2	1	1	2	2
2	1	2	1	1
2	1	1	2	2
2	1	2	1	1
2	2	2	2	2
1	2	2	2	2
2	2	1	2	1
2	2	2	2	2
2	1	2	2	2
1	2	1	2	2
2	2	1	2	2
2	2	1	2	1
2	1	1	1	1
1	1	2	2	2
2	1	1	2	2
1	1	2	2	2
2	2	1	2	2
2	1	1	2	1
2	1	1	1	1
2	2	1	2	2
2	2	1	1	1
2	2	1	2	1
1	1	2	1	1
2	1	2	2	1

2	2	2	2	2
1	1	1	1	1
2	2	1	2	1
2	2	1	2	2
2	2	1	1	1
2	2	2	2	2
2	2	1	1	1
1	1	2	1	1
1	2	1	1	2
2	2	1	1	1
1	2	2	1	1
1	1	1	2	2
1	1	1	1	1
2	1	1	1	1
2	2	1	2	2
1	2	2	1	1
2	2	2	1	1
1	1	1	1	1
2	1	2	2	1
1	2	2	1	1
2	1	2	2	2
2	2	2	2	2
1	2	2	2	2
2	2	1	1	1
2	2	1	2	2
2	2	1	1	1
2	2	2	2	2
2	2	1	2	1
2	2	1	1	1
2	2	2	2	1
2	1	1	2	2
1	2	1	1	1
2	1	2	1	1
1	2	2	2	2
1	2	1	2	2
2	1	2	1	1
1	2	2	1	1
2	1	2	1	1
2	1	1	2	2

2	2	2	2	2
1	2	1	2	2
1	2	2	2	2
2	1	1	1	1
1	2	1	2	1

Extrahepatic metastasis	AFP	N/L ratio	MTA	Therapeutic Efficacy
2	1	1	Naïve	SD
2	1	1	experienced	PR
2	2	1	Naïve	PD
2	2	1	Naïve	NE
2	1	2	Naïve	SD
1	1	1	experienced	SD
1	2	2	Naïve	SD
1	2	2	experienced	PR
1	1	2	experienced	SD
1	1	1	experienced	PR
1	1	1	experienced	SD
1	2	1	experienced	SD
1	2	1	experienced	PD
2	2	2	experienced	PD
1	2	1	experienced	PD
1	1	1	experienced	NE
1	1	2	Naïve	PR
1	1	1	experienced	SD
2	2	2	Naïve	NE
1	2	2	experienced	SD
2	1	2	experienced	SD
1	2	1	Naïve	SD
2	2	2	experienced	SD
1	2	2	experienced	SD
2	2	1	Naïve	PR
2	1	1	Naïve	SD
1	1	2	Naïve	NE
2	2	2	Naïve	CR
1	2	2	experienced	SD
1	2	1	Naïve	PR
2	2	2	Naïve	SD
1	1	1	Naïve	PR
1	1	2	experienced	NE
1	1	2	Naïve	PR
1	2	2	experienced	PD
2	1	1	experienced	SD
1	2	2	Naïve	PR
2	2	2	experienced	NE

1	2	1	Naïve	PD
1	1	1	Naïve	NE
2	1	2	Naïve	SD
2	2	1	Naïve	PR
1	1	1	Naïve	SD
1	2	2	Naïve	NE
2	1	2	experienced	PR
1	1	1	Naïve	PR
1	2	1	Naïve	SD
1	1	1	Naïve	PR
1	2	2	experienced	SD
1	1	2	Naïve	SD
1	2	1	Naïve	SD
1	1	1	Naïve	PR
2	2	1	Naïve	PD
1	2	2	experienced	NE
2	2	2	experienced	SD
1	1	2	Naïve	SD
2	1	2	experienced	NE
1	1	2	experienced	NE
1	2	1	Naïve	PR
2	1	1	Naïve	NE
2	2	2	Naïve	SD
1	1	1	Naïve	SD
1	2	2	Naïve	CR
1	2	1	Naïve	PD
1	2	2	Naïve	PR
2	1	1	Naïve	NE
1	2	2	Naïve	SD
2	1	2	Naïve	PD
2	2	2	Naïve	SD
1	1	1	Naïve	PR
1	1	1	experienced	PD
1	2	2	experienced	NE
2	2	2	Naïve	PR
1	2	2	experienced	NE
1	1	2	experienced	SD
1	1	1	experienced	SD
1	2	2	Naïve	PD

1	1	2	Naïve	SD
1	1	2	Naïve	SD
1	2	2	Naïve	PR
1	1	2	experienced	PR
2	1	1	Naïve	PD

Outcome	Survival time(w)
Alive	99.86
Alive	99.86
Alive	17.43
Alive	2
Death	92.43
Alive	97.86
Death	31.29
Alive	67
Alive	22.57
Alive	96.57
Alive	94.86
Alive	94.86
Death	18.29
Death	45.43
Alive	12.43
Alive	93.57
Alive	93.43
Alive	62.57
Death	11
Death	15.86
Death	30.86
Alive	24.57
Alive	31
Death	80.43
Death	33
Death	51.86
Death	5
Alive	80.71
Alive	78.57
Alive	76.71
Alive	73
Alive	74.86
Alive	74.86
Alive	73.86
Death	54
Alive	56.86
Death	36.14
Alive	72.29

Alive	17.29
Alive	69.86
Alive	69.43
Alive	67.86
Alive	68.86
Death	3.43
Death	35.14
Alive	59.57
Alive	58.57
Alive	58.29
Alive	60.43
Alive	60.86
Alive	55.86
Alive	55.71
Alive	6
Alive	36.29
Alive	29.14
Alive	53.43
Death	3
Death	10.71
Death	45.29
Death	6.57
Death	17.43
Alive	45
Alive	45.71
Alive	26.14
Alive	45.43
Death	8.57
Alive	40.86
Alive	19.57
Death	29
Alive	36.86
Alive	34.57
Alive	18.14
Alive	33.86
Alive	5.29
Alive	28.57
Alive	26.57
Alive	9.29

Alive	20.57
Alive	20.57
Death	10.57
Alive	22.57
Alive	10.57
